# Notes and News

**Published:** 1903-12-05

**Authors:** 


### Notes and News.

Princess Henry of Battenberg unveiled on Saturday a memorial tablet at the New Hospital for Women commemorating the endowment of a bed for the treatment of cancer. The funds have been raised by Miss Jenner in memory of the late Emperor and Empress of Germany, and only some £210 is now required to complete the endownment. A large number of supporters of the hospital were present at the ceremony, and speeches were delivered by Mrs. Fawcett, LL.D., Miss Cock, M.D., and the chairman of the Executive Committee, Mr. Henderson. The tablet, which is a beaten tin framed in oak, has been placed in the board room, and a small Doulton panel distinguishes the endowed bed.

The annual meeting of the Surgical Aid Society will be held on Monday, December 7th, at 4 o'clock, at Cannon Street Hotel.

The Right Hon. Walter Long recently opened a new infirmary for the Axbridge Workhouse. The building is of two storeys, with accommodation for 72 patients. The estimate was for £5,340, tendered by Mr. Charles Addicott, of Weston-super-Mare, the architect.

The London School of Tropical Medicine and the Royal Veterinary College of London have made mutual arrangements for the exchange of knowledge. Students of the school will be able to study the tropical diseases of animals at the college and veterinary surgeons will be able to study tropical medicine at the school.

Mr. Eustace Miles recently delivered a lecture at the West Heath School, Hampstead, so full of useful advice on the subject of the physical training of children that it is worthy the attention of all concerned in the care of the young. Mr. Miles wisely deprecated the use of long and monotonous exercises, and advocated an intelligent use of the muscles and short and brisk movements. He also advocated instruction in food values and the elements of cooking.

On Monday, December 7th, at 3 p.m., Sir Patrick Manson will deliver an address at the London School of Tropical Medicine, Albert Docks.

At the annual meeting of the Royal Scottish Hospital it was announced that a gift of £768 in Midland Railway stock had been received from the family of the late Mr. James Fraser for the purpose of creating a perpetual pension of £18, to be called the James Jessie Fraser pension.

The *Sun* newspaper is now under the proprietorship of Sir George Armstrong, Mr. J. S. Wood, of *The Gentlewoman*, and Mr. Madge, of *The Globe*. It has increased in size to eight pages, and will support Mr. Balfour's Government and Mr. Chamberlain's Fiscal Inquiry. We wish it all success in its new career.

At the recently held quarterly Court of the Middlesex Hospital a good report was made of progress made. The completion of the new offices, extension of the nursing home and of the nursing staff, and the satisfactory working of the new clinical and bacteriological laboratories were announced. The alterations and improvements have naturally entailed a large expenditure, and some £15,000 is needed to remove the deficiency.

Last week the Duke of Connaught presided at the festival dinner in aid of the Home and Hospital for Incurable Children, Maida Vale. The dinner was held at Princes' Restaurant, and a novelty was introduced by way of separate tables, for which parties had previously been arranged for friends of the institution and their guests. The idea appears to be a happy means of attracting attendance to hospital dinners, thus securing an interesting audience to the recitation of the work and claims of the charity.

The National Hospital for Consumption for Ireland, which some months ago was the recipient of the honour of Royal patronage, has now, with His Gracious Majesty's permission, adopted the prefix "Royal" to the corporate name. The hospital has for several years been carrying on most excellent work in the treatment of poor consumptives on the open-air principle. There are at present 67 beds in the Royal National Hospital, and the Board are adding a new wing for women patients, for whom additional accommodation is urgently needed.

A large portion of the sitting of the managers of the Metropolitan Asylums Board on Saturday was devoted to the consideration of the provision of sanatoria for consumptives by the Board. It was finally decided to request the Local Government Board to give an opinion as to whether it was desirable that the Asylums Board should undertake a scheme involving so large an expenditure and so great an increase in its duties. As Mr. A. C. Scorels pointed out during the discussion, the subject is surrounded with difficulties which should be carefully considered before a decision is arrived at, and the foremost question should be whether the accommodation should be for paupers or part-paying patients.

